# The Avalanche is Coming … And Just Now It's Starting to Snow

**DOI:** 10.3389/fimmu.2013.00165

**Published:** 2013-06-25

**Authors:** Richard Aspinall, Pierre Olivier Lang

**Affiliations:** ^1^Translational Medicine Research Group, Cranfield Health, Cranfield UniversityCranfield, UK; ^2^Clinique of Genolier, Nescens Centre of Preventive MedicineGeneva, Switzerland

Previous vaccine development has been driven mainly by policies and concerns around childhood, with the aim of preventing premature death amongst young children from infectious disease and stopping them from acting as carriers. Whilst this is important, the face of the world has changed recently and the problem has now to be extended to include the protection of a vulnerable aging population. Here we present the case for a need to develop a prophylactic regimen for older individuals which features vaccination at the center of a portfolio of treatments.

Advances in medical, social, and economic conditions have resulted in human population growth and an ever-extending life expectancy such that for the first time in our history the world population will soon have more people over the age of 65 than under the age of 5. The development of antibiotic therapy, progresses in vaccine technology combined with mass vaccination programs and government schemes to improve economic and social well-being mean that the majority of individuals over the age of 65 are currently physically more active than their counterparts a few decades ago (Michel et al., 2008). Moreover these individuals now travel more than either their parents or grandparents. The world is now so closely networked that any pathogen may spread across the globe within hours, as was observed with the recent H5N1 and H1N1 pandemics (Lang and Aspinall, 2012). In addition to the continuing trend toward increased life expectancy and the shifting demographics, the incidence and prevalence of chronic diseases are also expected to reach unprecedented levels as evidenced from a recent study on individuals over 80 years of age, which showed that many are living with an increasing number of comorbidities (Collerton et al., 2009).

## Influenza and an Aging Population

Influenza is a disease of viral origin commonly associated with a rapid onset of symptoms which may include fever, chills, fatigue, headache, joint pain, and nasal congestion. But the illness may be asymptomatic in many individuals facilitating the spread of the virus. In older individuals the most common presenting symptom may be a cough. The incidence of fever at presentation in older individuals is much less common than in younger individuals (McElhaney, 2005). Global estimates are that influenza infections cause between 250,000 and 350,000 deaths per year but that there are between 3 and 5 million severe cases per year (http://www.who.int/mediacentre/factsheets/fs211/en/). Influenza is commonly thought to be a disease of limited duration, but in older individuals it may lead to a period of prolonged bed rest. Because long periods of bed rest are associated with a loss of aerobic capacity (1% per week whilst bedridden), a loss of muscle capacity (5% per week), and a loss of bone density (∼1% per week) this may leave a previously healthy older individual as a weak frail person dependent on assistance from others for their normal activities of daily living (McElhaney, 2005). Moreover a common complication of influenza is secondary bacterial infection which greatly increases the likelihood of complications such as pneumonia and contributes to a poorer prognosis (Rothberg et al., 2008).

Influenza is mostly transmitted by aerosol, so infection is very dependent on social interaction. A diameter of ∼1 m around an individual is the area within which they can become infected by the airborne route (Lofgren, 2011). A potential second route of infection is through self-infection arising because many individuals frequently bring their hands in close contact with their faces. In humans face touching can occur up to almost 40 times per hour (Dimond and Harries, 1984). This action as a potential route of infection becomes important when one considers the survival of infectious organisms on inanimate surfaces such as handrails, door handles, or lift buttons. One recent study reviewed the survival of several infectious agents on seemingly dry inanimate surfaces and suggested that influenza could persist for 1–2 days on such surfaces (Kramer et al., 2006). From this it would appear that one of the greatest risks for becoming infected occurs during periods of close association with a number of individuals some of whom may be infected and the greatest likelihood of this occurring could be during periods of travel (Field et al., 2010). One distinct change between older individuals now compared with previous generations is that older individuals represent a substantial proportion of national and international travelers and might be at higher risk for some travel-associated diseases than younger individuals (Gautret et al., 2012).

## Influenza Vaccine for Older Individuals; Problems, and Perspectives

In many countries vaccination with the trivalent influenza vaccine (TIV) is recommended for all adults over the age of 60–65 to combat transmission (Michel et al., 2009). This vaccine contains three strains of inactivated influenza virus (influenza A H3N2 and H1N1 and influenza B) and whilst this immunization strategy has probably contributed to saving many lives, the exact magnitude of its benefits is still hotly debated (Lang et al., 2011). In terms of protection, studies have shown that TIV led to protection in more than 70% of younger individuals but was ineffective in over half of the elderly population (Hannoun et al., 2004; Monto et al., 2009; Lang et al., 2011). Greater resolution of this issue in the past has been attempted by holding randomized placebo controlled trials (Govaert et al., 1994), but this approach cannot be considered further, so the efficacy of the vaccine, especially in the elderly, has to be mainly derived from observational studies using data from research databases or health care utilization data systems (Lang et al., 2011).

The poor performance of the TIV in providing protection in older individuals has been known for some time, but to date there has been no satisfactory resolution of the issue. Several studies have been undertaken on possible reasons for this problem and these have produced a number of studies on age-associated changes in immunity which have identified problems with Langerhans cells (Shaw et al., 2011), dendritic cells (Panda et al., 2010), T cell function (Saurwein-Teissl et al., 2002), B cell function (Sasaki et al., 2011), T and B cell repertoires (Yager et al., 2008; Ademokun et al., 2011), innate immunity (Shaw et al., 2010), natural killer cells (Chidrawar et al., 2006), in addition to how all of these are affected by the nutritional status of the individual (Langkamp-Henken et al., 2006).

One of the major challenges for vaccine development in this population is associated with identifying measureable surrogate markers which are acceptable readouts of immune protection (Lang et al., 2011). Currently the widely accepted concept of protection is associated with post-vaccination antibody levels; a serum antibody hemagglutination inhibition (HI) titer ≥40 UI/L is the level associated with >50% reduction of the risk of developing influenza infection after exposure (Hannoun et al., 2004). But this correlate of protection was determined in healthy young individuals and not in older people with intercurrent comorbidities. In older adults other immune parameters actively contribute to protection so that HI titer alone may not guarantee immunity or predict susceptibility (Lang et al., 2011). This has led some authors to consider that no single marker should be considered as a surrogate of protection especially in a complex multicomponent system such as the immune system and that protection reflects the sum of various immune responses, including antibody and cell-mediated responses. So in this instance, poor responses to vaccine may result through the accumulation of multiple immune deficits (Lang and Aspinall, 2012).

## A Portfolio Approach

In a complex system such as the immune system with its multiple component overlaps, predicting individual responsiveness to influenza vaccination using a single method able to distinguish between a healthy and an immunosenescent state, would be very challenging. Especially as the ability of the immune system to respond to vaccination is further impinged upon by the impact of non-immune factors such as nutritional and metabolic status, and comorbidities from which adults increasingly suffer from as they age (Collerton et al., 2009).

The presence of these multiple issues associated with vaccine ineffectiveness underlines the degree of diversity which exists amongst older individuals, which in turn is somewhat expected since aging is neither regulated nor programed. Although each individual ages differently and in a way, which in part is dependent on lifestyle choices and environmental factors, there are common threads (Murabito et al., 2012). This may provide a route to a better understanding of method of protection following immunization in the future.

Since there are currently no tests associated with defining an individual's ability or inability to produce a protective response to vaccination, a more valuable approach would be to measure several aspects of the immune response following vaccination within an individual and compare these not only with known benchmark levels but also to confront these with validated biological and clinical outcomes. This would entail some degree of classification of older individuals into specific trends of aging on the basis of defined characteristics or their absence. In the past efforts have been made to achieve this and define an immune risk phenotype which includes T cell phenotypes, subset numbers, function, CMV status, and cognitive impairment (Wikby et al., 2005). Others have suggested that T cell receptor excision circles (TREC) levels and may be of interest in this classification (Mitchell et al., 2010). Once a characteristic is known to be outside the normal range it would be considered to be in “functional deficit” and the collection of deficits within the immune system which accrue with age would enable us to produce an accumulation of deficits score which could be correlated with clinical outcomes (Lang and Aspinall, 2012). These could assist us to classify individuals into clusters with common features. So, for example, a portfolio of treatments for immunosenescent individuals in the cluster characterized by low TREC levels who were also CMV positive could include therapy to rejuvenate output from primary lymphoid organs such as the use of interleukin 7 (Aspinall et al., 2007) whilst at the same time treating the herpes virus infection with a guanosine analog such as valaciclovir prior to vaccination. Use of such antivirals has provided some interesting results in mouse models. Treatment of elderly mice infected with CMV with valaciclovir, led to reduced influenza viral loads when they were subsequently challenged with influenza (Beswick et al., 2013). Alternatively individuals who are CMV negative and have both normal for age thymic output and T cell function but functionally underperforming dendritic cells may be recommended to receive the vaccine with additional adjuvant.

Such a move from the existing immunization menu toward a greater degree of personalized medicine could contribute to accelerating the development of new vaccines with higher efficacy and of specific combined therapeutic approaches than those currently available.
